# SARS-CoV-2 Spike Protein Unlikely to Bind to Integrins *via* the Arg-Gly-Asp (RGD) Motif of the Receptor Binding Domain: Evidence From Structural Analysis and Microscale Accelerated Molecular Dynamics

**DOI:** 10.3389/fmolb.2022.834857

**Published:** 2022-02-14

**Authors:** Houcemeddine Othman, Haifa Ben Messaoud, Oussema Khamessi, Hazem Ben-Mabrouk, Kais Ghedira, Avani Bharuthram, Florette Treurnicht, Ikechukwu Achilonu, Yasien Sayed, Najet Srairi-Abid

**Affiliations:** ^1^ Sydney Brenner Institute for Molecular Bioscience, Faculty of Health Sciences, University of the Witwatersrand, Johannesburg, South Africa; ^2^ Laboratory of Biomolecules, Venoms and Theranostic Applications, LR20IPT01, Institut Pasteur de Tunis, University of Tunis El Manar, Tunis, Tunisia; ^3^ National Gene Bank of Tunisia, Boulevard du Leader Yesser Arafet, Tunis, Tunisia; ^4^ Université de Tunis El Manar, Institut Pasteur de Tunis, LR11IPT08 Venins et Biomolecules Therapeutiques, Tunis, Tunisie; ^5^ Laboratory of Bioinformatics, Biomathematics and Biostatistics (BIMS), Institut Pasteur de Tunis (IPT), University of Tunis El Manar, Tunis, Tunisia; ^6^ Department of Virology, National Health Laboratory Services and the School of Pathology, University of the Witwatersrand, Johannesburg, South Africa; ^7^ Protein Structure-Function Research Unit, School of Molecular and Cell Biology, University of Witwatersrand, Johannesburg, South Africa

**Keywords:** SARS-CoV-2, COVID-19, integrin, RBD, molecular dynamcis

## Abstract

The Receptor Binding Domain (RBD) of SARS-CoV-2 virus harbors a sequence of Arg-Gly-Asp tripeptide named RGD motif, which has also been identified in extracellular matrix proteins that bind integrins as well as other disintegrins and viruses. Accordingly, integrins have been proposed as host receptors for SARS-CoV-2. However, given that the microenvironment of the RGD motif imposes a structural hindrance to the protein-protein association, the validity of this hypothesis is still uncertain. Here, we used normal mode analysis, accelerated molecular dynamics microscale simulation, and protein-protein docking to investigate the putative role of RGD motif of SARS-CoV-2 RBD for interacting with integrins. We found, that neither RGD motif nor its microenvironment showed any significant conformational shift in the RBD structure. Highly populated clusters of RBD showed no capability to interact with the RGD binding site in integrins. The free energy landscape revealed that the RGD conformation within RBD could not acquire an optimal geometry to allow the interaction with integrins. In light of these results, and in the event where integrins are confirmed to be host receptors for SARS-CoV-2, we suggest a possible involvement of other residues to stabilize the interaction.

## 1 Introduction

The molecular mechanism of human infection with SARS-CoV-2 has been studied extensively (Shang et al., 2020a; Harrison et al., 2020; Li et al., 2021). Alveolar epithelial cells are thought to be the main target for the virus. Indeed, in pioneering work, Chu *et al.* (Chu et al., 2020), studied the tropism of SARS-CoV-2 by inoculating it into 24 cell lines covering seven organs and tracts. They found that the virus most efficiently replicates on lung-type cell lines. Other organs can also be targeted including intestinal tracts, liver, and kidney (*idem*). At the molecular level, the interaction with the host cell involves primarily the homotrimeric spike protein (S protein) expressed on the virus surface. Prior to cell attachment, the spike protein arranges its three Receptor Binding Domains (RBD) in a laying-down configuration, which could help to evade the immune system (Berry et al., 2004). Human viruses frequently use mammalian cell surface receptors to attach and to enter host cells (Sheppard, 2003). During the interaction process with the host cell, the spike protein switches one of the RBD domains to a standing-up configuration, thus exposing the Receptor Binding Motif (RBM) to the interaction surface of the Angiotensin-Converting Enzyme 2 (ACE2) receptor. ACE2 is widely regarded as the main entry point for the virus to the cellular machinery of the host (Othman et al., 2020; Wan et al., 2020). However, evidence suggests the possibility of other receptors and co-receptors that might be as relevant as ACE2. The proteomic analysis that helped to establish the interactome map, suggested the putative implication of more than 300 host proteins in the interaction with SARS-CoV-2 (Gordon et al., 2020). While many of these proteins are expected to be false-positive hits, other studies have pointed out the critical role of specific host proteins and macromolecules as co-receptors (Zamorano Cuervo and Grandvaux, 2020), such as neuropilin-1 (Cantuti-Castelvetri et al., 2020), heparan sulfate (Clausen et al., 2020), sialic acids (Qing et al., 2020), CD147 (Aguiar et al., 2020) and GRP78 (Ibrahim et al., 2020). Recently, Sigrist et al. (2020) have identified an Arg-Gly-Asp (RGD) motif in the sequence of the spike RBD which is found to be exposed at the surface of the interaction domain. This motif was originally identified within the extracellular matrix proteins, including fibronectin, fibrinogen, vitronectin, and laminin that mediate cell attachment. Integrins are membrane proteins that act as receptors for these cell adhesion molecules via the RGD motif (Hamidi et al., 2016). Three main integrins expressed on airway epithelial cells were described to play an important role in virus infection (Isberg and Tran Van Nhieu, 1994). *α*_2_ *β*_1_, a collagen and laminin receptor, play a critical role in cell infection by echovirus (Eisner, 1992). Based on these findings, Sigrist et al. (2020) concluded that integrins can also interact with the spike protein. Several other studies have built on this hypothesis to support the role of integrins as spike protein receptors (Luan et al., 2020; Beddingfield et al., 2021; Dakal, 2021) and to exploit the property for potential therapeutic applications (Yan et al., 2020). Moreover, Beddingfield et al. (2021) showed, by *in vitro* analysis, that the interaction with integrins is a plausible hypothesis. Integrins are heterodimeric receptors that interact favorably with the extracellular molecules by forming a cleft at the protein-protein interface between the beta-propeller and a beta1 domains from the alpha and beta subunits (Xiao et al., 2004). The cleft contains the Metal Ion-Dependent Adhesion Site (MIDAS) harboring an Mg^2+^ ion. Differential expression of *α*_2_ *β*_1_, *α*_3_ *β*_1_, *α*_4_ *β*_1_, *α*_5_ *β*_1_, *α*_7_ *β*_1_, *α*_6_ *β*_4_, *α*_9_ *β*_1_, *α*_*V*_ *β*_5_, *α*_*V*_ *β*_6_, *α*_*V*_ *β*_8_ integrins was revealed in human lung cells (Weinacker et al., 1995; Cambier et al., 2000; Bazan-Socha et al., 2005). Indeed, *α*_2_ *β*_1_, *α*_3_ *β*_1_, *α*_6_ *β*_4_, *α*_9_ *β*_1_, *α*_*V*_ *β*_5_, *α*_*V*_ *β*_6_ and *α*_*V*_ *β*_8_ are expressed in airway epithelial cells, which are the main target of coronavirus (Ravindra et al., 2020). Among these, only *α*_*V*_ *β*_5_, *α*_*V*_ *β*_6_ and *α*_*V*_ *β*_8_ can recognize RGD motif while *α*_5_ *β*_1_ integrin was not shown to be expressed in healthy epithelial cells (Sheppard, 2003). The activity of integrins can be inhibited by disintegrin peptides purified from animals such as snakes, scorpions and insects. The majority of these disintegrins incorporate an RGD motif in their sequences (Gasmi et al., 2001; Olfa et al., 2005; Bazaa et al., 2007; Assumpcao et al., 2012; Ben-Mabrouk et al., 2016). Most of the arguments about the validity of the RGD motif in SARS-CoV-2 RBD as an interacting segment with integrins are supported by sequence-based and structural-based analysis. However, the microenvironment of RGD imposes a critical steric hindrance that could prevent the RBD from optimally interacting with integrins. To investigate the extent of such effect on the RGD/RBD conformational and binding properties, we conducted a computational study involving microscale accelerated molecular dynamics simulation and protein-protein docking.

## 2 Methods

### 2.1 Structural Data

All the structures with complete 3D coordinates of the RBD were explored. They include X-ray crystallography and the cryo-electron microscopy structures. The coordinates of the RBD domain were extracted from the entries of the complete spike protein. In total, we obtained 90 Protein Data Bank (PDB) files (Supplementary Data S1).

### 2.2 Normal Mode Analysis

The normal mode analysis (NMA) approach represents an efficient and powerful tool for predicting and characterizing the large-scale conformational transitions in protein structures around their equilibrium fluctuation. For this study, the Bio3D package in R (version 2.4-1.9000) was utilized to conduct a comparative NMA analysis of a large ensemble of structures (Fuglebakk et al., 2012). All atoms low-frequency normal modes were calculated under the coarse-grained Elastic Network Model (ENM). Prior to the calculation, structures were aligned to an invariant region of RBD residues. Root Mean Squared Inner Product (RMSIP) was computed from the corresponding eigenvectors of the normal modes to calculate a score quantifying the overlap between modes. The RMSIP was calculated between all the pairs of RBD structures from the collected ensemble of PDB files.

### 2.3 Accelerated Molecular Dynamics

**FIGURE 1:**
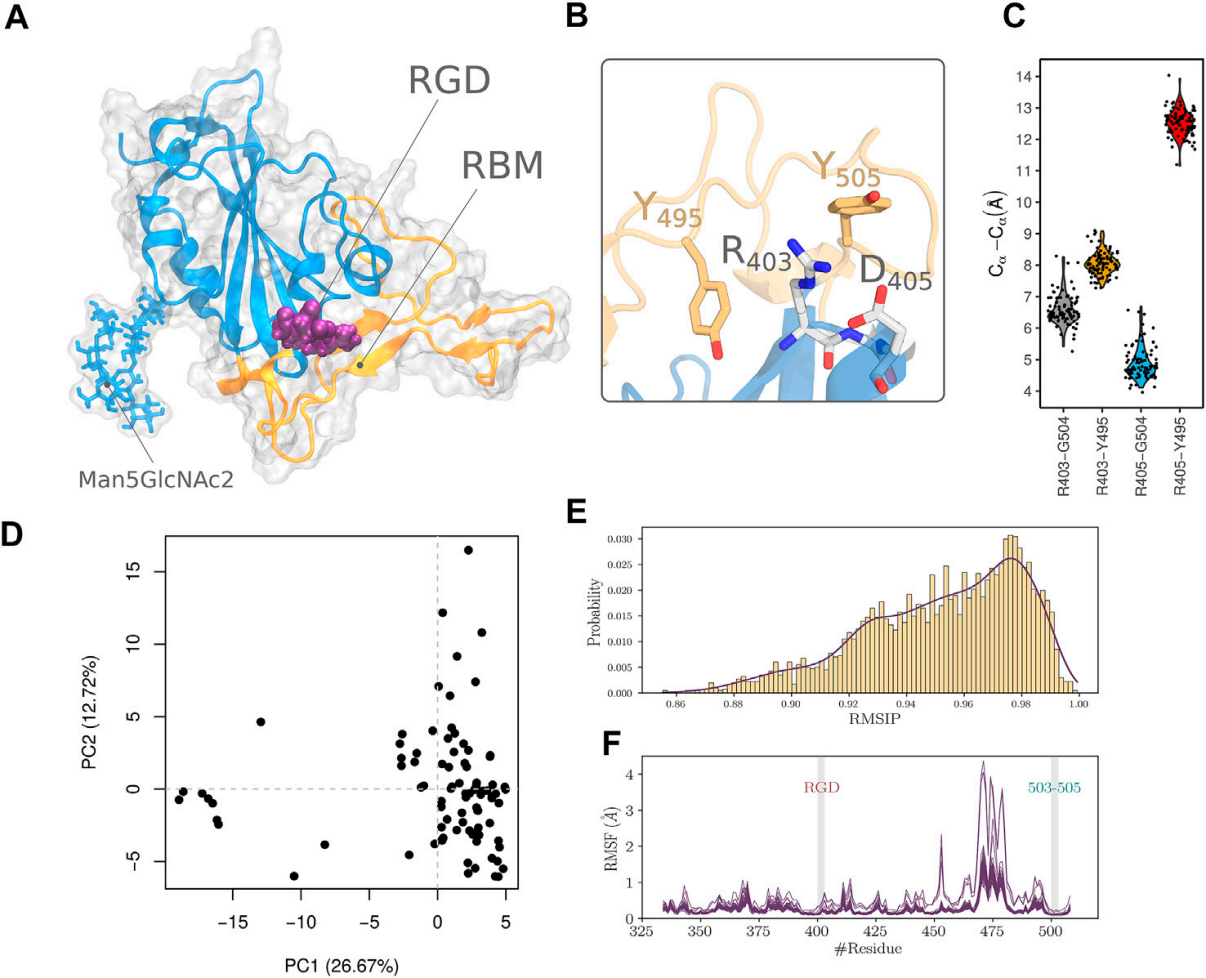
Analysis of the RBD structural ensemble. **(A)** Structure of RBD showing the RGD motif, the Man5GlcNAc2 polysaccharide and the RBM segment. **(B)** Arrangement of the RGD motif relative to residue Y505 and Y495. **(C)** Statistical measurements of distances between RGD residues and D405 and Y495 collected from the ensemble of experimental structures. **(D)** Projection of RBD structure in the PC1-PC2 subspace of the PCA performed on pre-aligned and superimposed ensemble of structures. **(E)** RMSIP density plot calculated using the normal modes of each pair of structures of the ensemble. **(F)** RMSF profile of all the structures in the ensemble computed from all atoms normal mode analysis.

Accelerated molecular dynamics (aMD) enhances the sampling of a protein conformational space by lowering energy barriers of the energy landscape (Hamelberg et al., 2004). A bias term is added to the potential energy *V*(*r*) when the value falls below a certain threshold as follows:

$$V^{*}\left(r\right)=V\left(r\right)+\Delta V\left(r\right)$$

$$\Delta V\left(r\right)=\left\{\begin{array}{cc} \; & 0\text{ if }V\left(r\right)>E\\ \; & \frac{{\left(E-V\left(r\right)\right)}_{2}}{\alpha +E-V\left(r\right)}\text{ if }V\left(r\right)<E \end{array}\right.$$

where Δ*V*(***r***) is the bias; *V*(***r***) is the potential energy calculated from the vector of coordinates ***r*** of all the atoms in the system; *E* is the threshold value of the energy, and *α* is the acceleration factor (Wang et al., 2011). We used the crystal structure of SARS-CoV2 RBD in complex with H11-D4 antibody (PDB code 6YZ5) at a resolution of 1.8 Å to conduct the simulations. Parameters from the ff14SB force field (Maier et al., 2015) were assigned to the atoms of the system using AMBER molecular dynamics simulation package, version 18 (Case et al., 2018). After removing the antibody and the heteroatoms from the structure, we built a Oligomannose-5 glycan (Man5GlcNAc2) type polysaccharide structure and linked it covalently to residue N343 of the RBD (Figure 1A). The topology of the glycan was identified to be the major form for this amino acid (Watanabe et al., 2020). The system was then neutralized, and TIP3P water molecules were added to a truncated octahedron simulation box where the edges are at a minimum distance of 12 Å for any atom of the solute. Three stages of energy minimization were used to clean the geometry of the atoms and to relax the system. First, we used 5,000 steps of steepest descent minimization followed by 15 ,000 steps of conjugate gradient minimization while restraining both water and protein atoms at their initial positions using a force constant of 100 kcal/mol/Å^2^ and a non-bonded contact cutoff of 12 Å. We then applied the same minimization series with 400 steps of the steepest descent algorithm and 9,600 steps of the conjugate gradient algorithm while applying the constraining force on the protein atoms only. At the final stage, we ran the same cycle and we only lowered the constraining force constant to 0.1 kcal/mol/Å^2^ applied to the protein atoms. To further relax the system, we applied a heating stage of molecular dynamics by increasing the temperature from 50 to 300 K while maintaining a force constant of 10 kcal/mol/Å^2^ on the heavy atoms of the RBD. A Langevin thermostat with a collision frequency of 5 ps^−1^ was applied to control the temperature fluctuation. Following the heating stage, we lifted the constraining forces gradually by an increment of 1 over 11 intervals of 100 ps. The restrained molecular dynamics were run in the NPT ensemble by maintaining the pressure at 1 atm using a relaxation time of 2 ps. The SHAKE method was applied for all the stages of the simulation to constrain the bonds involving hydrogen atoms which allowed an integration time of 100 fs. The Particle Mesh Ewald method was applied to calculate the electrostatic forces. The production phases were run under the NVT conditions. To calculate the different parameters for the aMD simulation, we first run classical molecular dynamics for a total time of 100 ns. From there, we estimated the values of the parameters to calculate the boosting term. The aMD simulation was run in 3 independent replicates for a total time of 1 µs each. An extra boost to the torsional space was added, and the trajectory was constructed by collecting the snapshots at every 10  ps of the running simulation.

### 2.4 Molecular Dynamics Data Analysis

The crystal structure was set as a reference conformation. Analysis of the molecular dynamics trajectory was made with an in-house python code. Principal Component Analysis (PCA) (David and Jacobs, 2014) was calculated for all heavy atoms in the protein, which allowed the detection of dynamical patterns with functional relevance. The translational and rotational related dynamic was first removed by fitting the ensemble of snapshots to the crystal structure of RBD. The low dimension components were calculated to return the corresponding eigenvalues and eigenvectors as well as the projection of the atomic coordinates into the lower-dimensional subspace. Clustering analysis was executed using a hierarchical algorithm embedded in the “cpptraj” analysis tool implemented by AMBER. In this regard an *ϵ* cutoff of 2 Å was used. To assess the convergence of the simulation, the cumulative number of clusters (*CNC*) as a function of time and the evolution of informational entropy (*H*) were calculated. The informational entropy is defined by the following formula.

$$H=-\overset{n}{\underset{i=1}{\sum }}p_{i}\log\left(p_{i}\right)$$

*p*_*i*_ is the probability of the *i*th found cluster, as a function of simulation time. To recover the unbiased free energy landscape from the ensemble of conformations sampled by aMD, we reweighted the probability sampling landscape according to the following equation.

$$v_{i}=k_{\text{b}}TLn{\left[\frac{P\left(x_{i}\right)}{P_{\text{max}}\left(x\right)}\right]}^{2}$$

*k*_*b*_ is the Boltzmann constant, T was set to 298 K, *P*(*x*_*i*_) estimates the probability of a conformational event obtained by binning along the reaction coordinate using the histogram method. The number of bins was set to 50. *P*_max_(*x*) is the maximum probability of the discrete state.

### 2.5 Protein-Protein Docking

Protein-protein docking was run using the prediction interface of HADDOCK2.2 web server (van Zundert et al., 2016). Integrin structures of *α*_5_ *β*_1_, *α*_IIb_ *β*_3_, and *α*_*V*_ *β*_8_, corresponding to PDB entries 3VI4, 3ZDY and 6UJC respectively, were defined as receptors. The structure of integrins is in a bound state with an RGD binding segment which was removed before running the docking. All residues within a 7 Å distance from the bound RGD in the integrin structure were used to define the active residues of the receptor. Multiple conformations of RBD, compiled from the molecular dynamics simulation, were employed as ligand structures to run the cross-docking. The amino acids of the RGD motif (in position 403-405) were used to define the active residues of the ligand structures. All other parameters of HADDOCK2.2 were kept to their default settings. The structure of the most populated cluster for each docking run was selected for analysis.

## 3 Results

We explored the crystal structure of RBD (PDB code 6YZ5). The RGD motif extends over residues 403-405. R403 is located at the C-terminal end of the fourth *β*-strand of the RBD, while both G404 and D405 are part of its *α*-helix (Figure 1A). We noticed that only D405 and the guanidinium group of the R403 side chain are solvent-exposed (Figure 1B). RGD motif shows a considerable kink defined by the main chain atoms and the *C*_*β*_ atoms of R403 and D405. Such configuration leads to the close contact between the RGD motif charged groups with a distance of 4.1 Å. This conformation is different from the optimized configuration of integrin interacting RGDs that adopt an extensive or a slightly kinked configuration (Kapp et al., 2017). The conformation might be imposed, in part, by the tight interactions with nearby amino acids of the RBD that include Y495 and Y504 (Figure 1B). Both residues are part of the receptor-binding motif with ACE2 (Shang et al., 2020b). We, therefore, hypothesized that in order to come to an integrin-compatible conformation for RGD, the nearby segments incorporating Y495 and Y504, have to move outwardly relative to the motif. We first attempted to detect such an event in the collected dataset, by assuming that a functionally relevant conformation, could be sampled in the large number of RBD experimentally solved structures. We thought that measuring the distance between reference amino acids in the RGD segment, i.e. R403 and D405, and other residues in the nearby RBM amino acids (Y495 and G504) might be a good proxy to evaluate the extent of the outward movement of the latter segment relative to RGD. The results of this analysis are reported in (Figure 1C). The median distances are 6.4, 8.0, 4.7, and 12.5 Å, corresponding respectively to R403-Y504, R403-Y495, D405-Y504, and D405-Y495 pairs of residues. The distances also show low variability with a maximum difference between the upper and lower values of 2.7 Å noticed for the D405-Y504 pair of residues.

### 3.1 Normal Mode Analysis

Previous work (Bahar et al., 2010; Bende, 2010) showed all-atoms elastic network normal mode analysis to be successful in describing the collective dynamics of a wide range of biomolecular systems. We therefore analyzed the ensemble of experimental RBD structures to verify the extent of conformational remodelling that can be adopted and whether it can lead to a better configuration of the RGD atoms in order to be able to interact with integrins. We performed a PCA on the pre-aligned and superimposed ensemble of structures. Data along PC1 and PC2 are relatively clustered in the lower right corner of Figure 1D, except for a few structures that showed the highest values of PC2 or the lowest values of PC1. Particularly for these structures, this might indicate divergent structural properties compared to the other members of the dataset. To proceed with a quantitative and more objective comparison, we calculated the RMSIP to assess the degree of overlap of the normal modes between the members of the constructed ensemble as proposed in related work (Yao et al., 2016). A score of 0.70 is considered a good correspondence, while a score of 0.50 is considered fair (Amadei et al., 1999). We found that the RMSIP values are ranged from 0.86 to 1 (Figure 1E) which shows a high level of similarity and agrees with the results from the PCA calculated from the normal modes. We also evaluated the structural deformation adopted by the RBD in terms of Root Mean Square Fluctuation (RMSF) calculated from the projection of the normal modes (Figure 1F). The structures of the ensemble show an overall similar profile of residue fluctuations in almost all except for some, where increasing flexibility by the amino-acids of the RBM segment is noticed. Furthermore, we noticed that the lateral chains of segment 503-505 residues (we refer to this cluster of residues as C1) are the closest residues from RBM that interact with the RGD segment. This was also detected from the distance calculation shown in Figure 1C. We thought that these residues are critical in controlling the conformational properties of the RGD segment. However, The RMSF profile revealed limited flexibility for both RGD motif and 503-505 segment showing a maximum displacement of 0.2 Å.

### 3.2 Accelerated Molecular Dynamics Shows Local Flexibility Mainly in the Receptor Binding Motif Segment but Not in RGD Microenvironment

**FIGURE 2:**
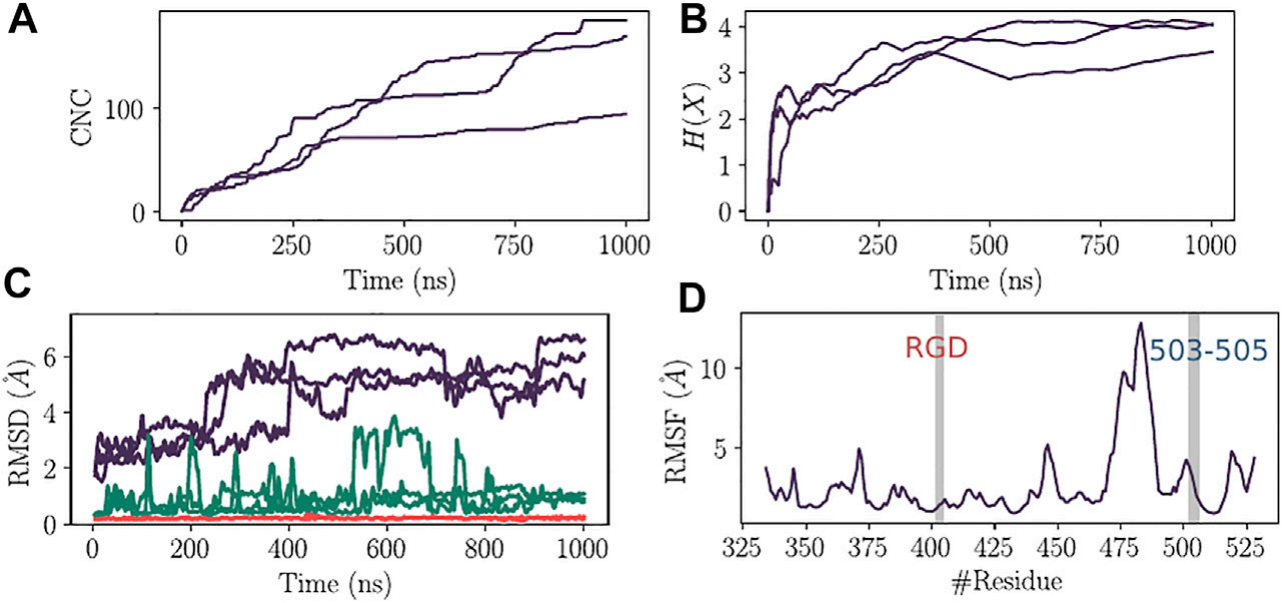
Convergence analysis of aMD and structural deviation of RBD. **(A)** Cumulative number of clusters as a function of time for the three replicas of aMD trajectories. **(B)** Evolution of the Shannon’s entropy (*H*(*X*)) for the three replicas of aMD trajectories. **(C)** Root Mean Square Deviation of RBD structure (Purple), C1 cluster of residues (Green) and the RGD motif (Red). **(D)** Root Mean Square Fluctuation of RBD residues calculated for the *C*_*α*_ atoms from the combined aMD trajectories.

Three independent aMD simulations were conducted for a total simulation time of 3 µs. This allows for efficient sampling of the energy landscape for SARS-CoV-2 RBD. The utility of aMD has been previously shown in many macromolecular systems including G-protein coupled receptors, bovine pancreatic trypsin inhibitor, and *α*-1-Antitrypsin (Duan et al., 2019). The main goal of this analysis was to identify the most populated conformations that the RBD can take to exert its function of interacting with the host receptors. In the event that the virus binds to integrins via the RGD motif, we would be able to detect a conformational state adapted for such interaction within the set of the sampled aMD snapshots. First, to assess the convergence of the different independent simulations, we calculated the cumulative number of the detected conformational clusters as well as the evolution of Shannon’s entropy (Figures 2A,B). We found that, except for one run, all the trajectories show adequate convergence starting from 300 ns in terms of CNCs. The entropy value also converged for all the replicates around 300 ns (Figure 2B). The coverage of the conformational landscape for RBD was therefore reasonable in the context of our research question. We then verified the conformational drift from the initial structure of RBD for the total *C*_*α*_ atoms, the *C*_*α*_ of the RGD segment, and those of both RGD motif and the C1 cluster that harbors the Y504 residue (Figure 2C). The latter was included given its proximity to RGD as well as the presumed role that it may play to control the structural properties of the motif. Based on all residue Root Mean Square Deviation (RMSD) values, that can exceed 6 Å, RBD might adopt a significant conformational arrangement. However, the RGD motif does not seem to share this property as the range of RMSDs is less than 0.5 Å. In addition, the C1 residues also did not show a large conformational drift compared to the crystal structure since the corresponding RMSD values are mostly below 2.5 Å. This indeed can also be seen from the RMSF profile of the *C*_*α*_ atoms of RBD (Figure 2D). The region that shows the largest flexibility corresponds roughly to the RBM residues. The RGD motif shows RMSF values of less than 2 Å while the loop 503–505 has a maximum value of 3.1 Å.

### 3.3 Principal Component Analysis and Clustering Analysis Show No Major Conformational Change in RGD and Its Microenvironment

**FIGURE 3:**
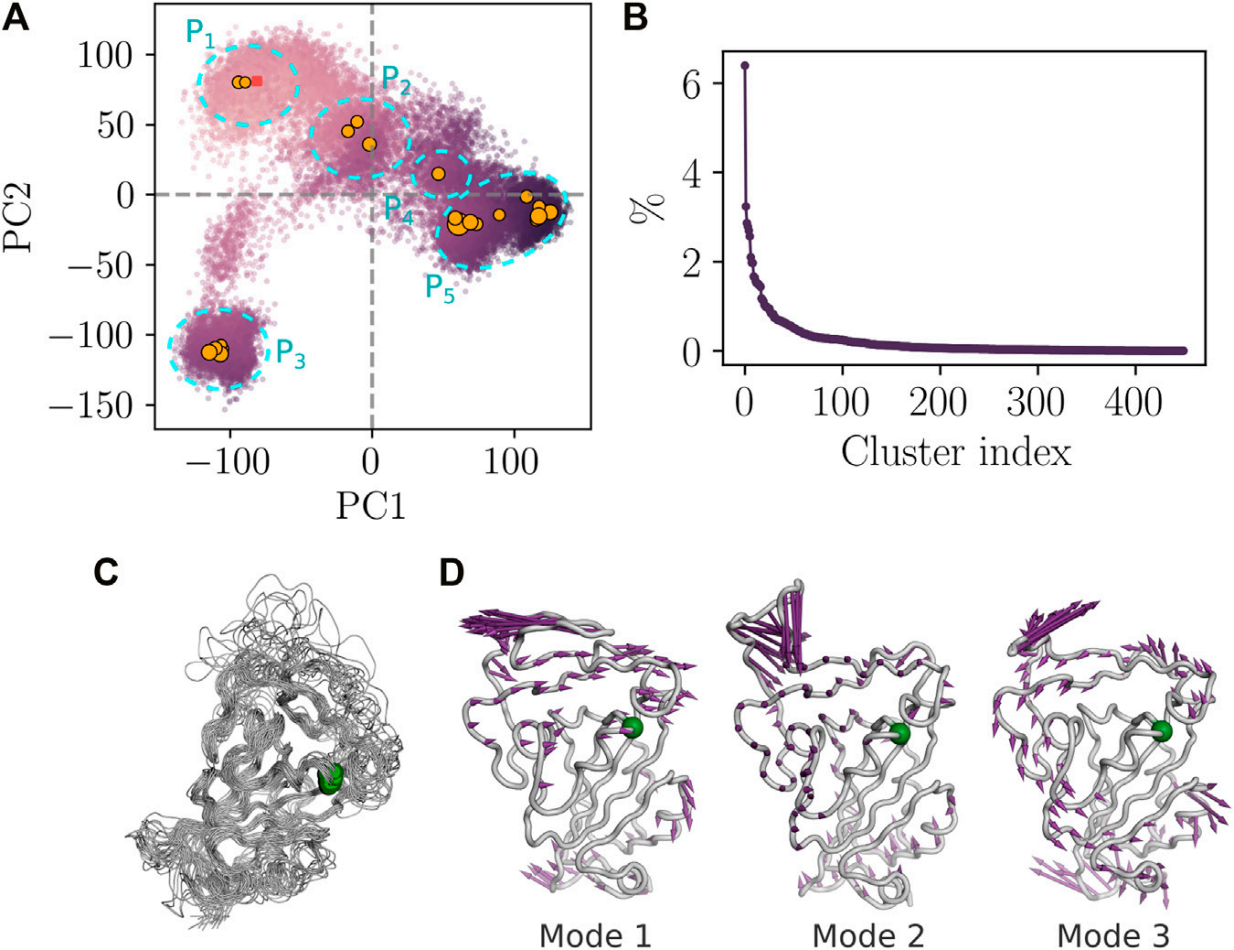
Essential dynamics of RBD from aMD simulation. **(A)** PCA analysis from the combined replicas. The color of the dots varies as a function of the structural deviation (RMSD) to the crystal structure of RBD. i.e, light purple color indicates lesser deviation and dark purple indicates higher values of RMSD. The square point corresponds to the projection of the crystal structure onto the first and the second subspaces. Orange circles correspond to the centroids of the highly populated clusters and the size of the circles is proportional to the occupancy of the cluster. **(B)** Occupancy of RBD structural clusters. **(C)** Structural alignment of the highly populated clusters (occupancy >1%). Green spheres indicate the position of the RGD motif. **(D)** Porcupine plot corresponding to projections of *C*_*α*_ atoms onto the first three non-rotational and non-translational normal modes.

We have conducted a principal component analysis using the total set of conformations from the three combined independent trajectories. The protein heavy-atom coordinates were projected onto the subspaces defined by the first and the second components. The aMD simulation was capable of capturing different states of the RBD. We noticed that the structure drifted considerably from the initial crystal structure (red rectangle in Figure 3A), thus demonstrating the convenient sampling of the RBD phase space that allows ascending the energy barriers. Clustering analysis focused on the clusters showing more than 1% of occupancy. Twenty one major clusters were detected of which the highest-ranked member shows the occupancy of 6.3% (Figure 3B).

Essentially, the PCA plot can be subdivided into five different partitions according to the density of the major conformational clusters (Figure 3A). P1 partition consists of the structures that are close to the bound conformation of RBD. Partitions P2 and P4 correspond to transition states with lower occupancies compared to the other partitions. P3 and P5 correspond to highly populated partitions where the density of the projected atom coordinates is high as shown from the large number of major clusters agglomerated together in the PCA plot. Highly populated partitions, i.e. P1, P3, and P5, may describe the three relevant discrete functional states of RBD corresponding to the bound, up and down states (Henderson et al., 2020). However, we were unable to verify this, given that the experimental structure of these states lack the atomic details in some RBD segment regions and those at close proximity to subdomain-1 of the spike protein. Nevertheless, the free energy landscape based on PC1 and PC2, as reaction coordinates established after correcting for the biased sampling of aMD, shows indeed that P2, P3, and P5 correspond to minimum energy wells on the one hand and confirms that P2 and P4 partitions describe transition states on the other hand (Supplementary Data S2). Superposition of the representative structures of the highly populated clusters revealed a rigid core of the RBD that harbors the RGD motif of low flexibility (Figure 3C). Porcupine plots, depicting the direction and the amplitude of motion across the three non-rotational and non-translational normal modes, also highlight the location of the RGD motif within a rigid core of the RBD, characterized by a low amplitude displacement vector (Figure 3D). Moreover, the RGD motif is rigid in modes 2 and 3, while it moves in the same direction of the segment 503-505 in mode 1.

### 3.4 Favorable Geometrical Features for the Interaction Between RGD and Integrins Are Not Sampled in the Receptor Binding Domain Ensemble

**FIGURE 4:**
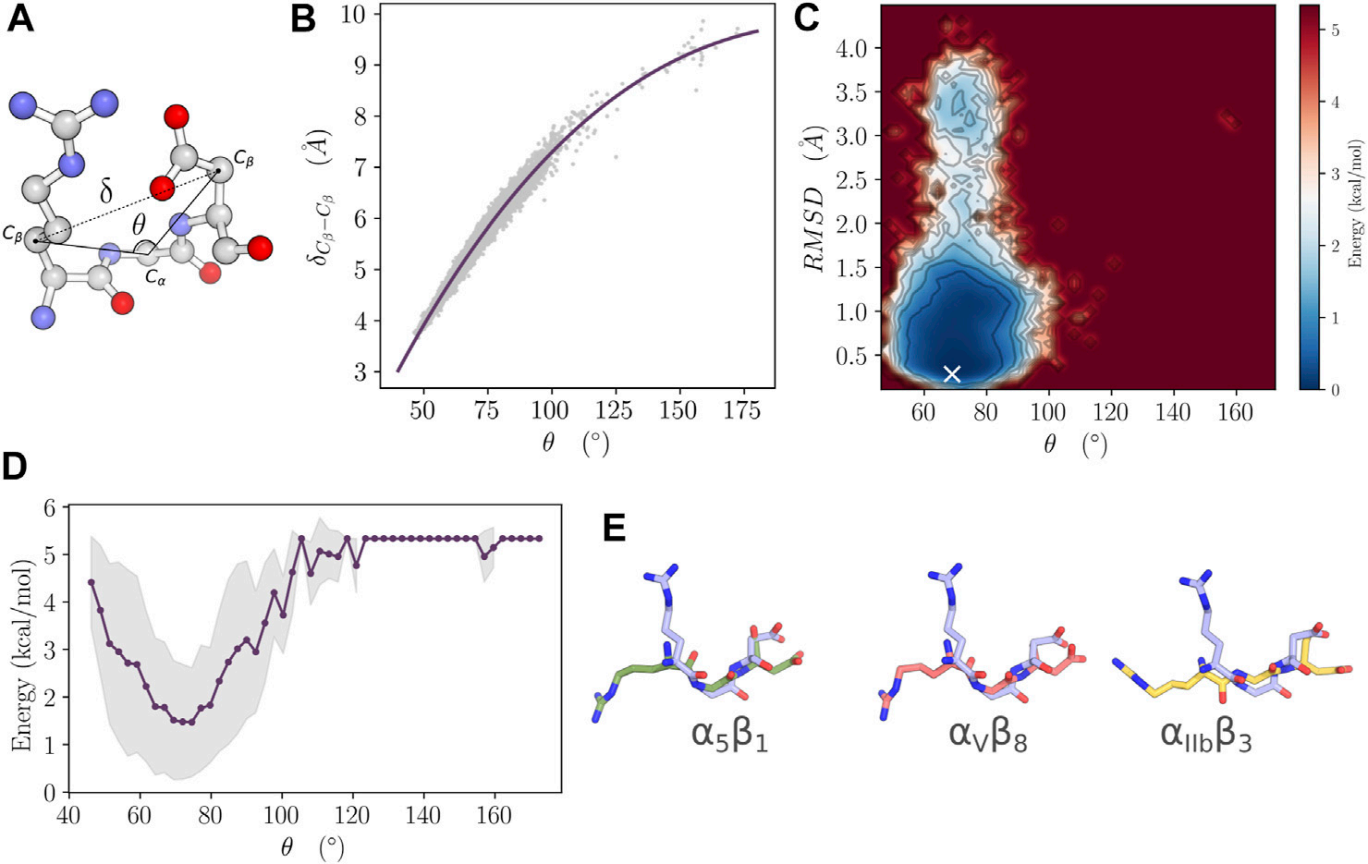
Free energy landscape analysis of the RBD. **(A)** $\delta_{C_{\beta }-C_{\beta }}$ distance and the *θ* angle are indicated on the structure of the RGD segment from RBD. **(B)** Correlation of $\delta_{C_{\beta }-C_{\beta }}$ and *θ*. Data were fitted to a polynomial model (*R* ^2^ = 0.97). **(C)** Free energy landscape as a function of *θ* and the RMSD of the C1 residue cluster. The white marker indicates the position of the global minimum. **(D)** Variation of the energy as a function of *θ*. The gray shading indicates the boundaries defined by the standard deviation of the energy averaged along the reaction coordinate. **(E)** The RGD structure corresponding to the minimum of energy (light blue) was fitted and compared to the RGD structure in its bound form with *α*_5_ *β*_1_ (Green), *α*_*V*_ *β*_8_ (light pink) and *α*_IIb_ *β*_3_ (Yellow) integrins.

Previous research using RGD peptide analogs suggested that extended conformation, spanning the atoms of the aliphatic side chain of Arg and Asp residues as well as the atoms of the main chain of RGD, has to take place to be capable of interacting with integrins (Civera et al., 2017; Kapp et al., 2017). Moreover, the distance between the *C*_*β*_ atoms of Arg and Asp must be within a range of 7 Å to 9 Å. To examine if these properties occurred during aMD simulation, we calculated the angle described by the *C*_*β*_, *C*_*α*_, *C*_*β*_ of R403, G404, and D405 residues, respectively, allowing to assess the level of extension (Figure 4A). We also calculated the distance between the *C*_*β*_ atoms of R403 and D405. $\delta_{C_{\beta }-C_{\beta }}$ and *θ* describe a wide range of values of 3.6 Å to 9.8 Å and 46° to 172°, respectively (Figure 4B). However, the data are skewed towards the lower end of the value ranges. Roughly, *θ* has more density in the 46° to 110° range, while the proportion of $\delta_{C_{\beta }-C_{\beta }}$ is ranging in higher values of 3.8 Å to 7.7 Å. A strong correlation was also noticed between $\delta_{C_{\beta }-C_{\beta }}$ and *θ* with an *R* ^2^ value of 0.97 when we fitted the data to a polynomial model. Therefore, we choose the *θ* angle and the RMSD of the C1 cluster of residues as reaction coordinates (Figure 4C). The FEL has a single highly populated minimum where the values of *θ* roughly span a range of 58° to 83° while the RMSD is low and does not exceed 1.5 Å. Averaging the energy over the binned values of *θ* shows a depth in the energy well of around 3 kcal/mol (Figure 4D). It also reveals that the more extended *θ* is in the less favorable energy. Indeed the conformation with the lowest energy value shows a significant divergence compared to the states of the RGD motif in its bound form with *α*_5_ *β*_1_, *α*_IIb_ *β*_3_ and *α*_*V*_ *β*_8_ integrins (Figure 4E). *θ* and $\delta_{C_{\beta }-C_{\beta }}$ for the lowest energy conformation were measured to 67° and 5.4 Å, respectively. The RGD motif however, clearly adopts an extended conformation in its bound form as revealed by *θ* values of 146°, 173° and 145° and $\delta_{C_{\beta }-C_{\beta }}$ values of 8.9, 9.6 and 8.9 Å for *α*_5_ *β*_1_, *α*_IIb_ *β*_3_ and *α*_*V*_ *β*_8_ respectively.

### 3.5 Protein-Protein Docking Shows the Inability of RGD Motif to Interact With Integrins

**FIGURE 5:**
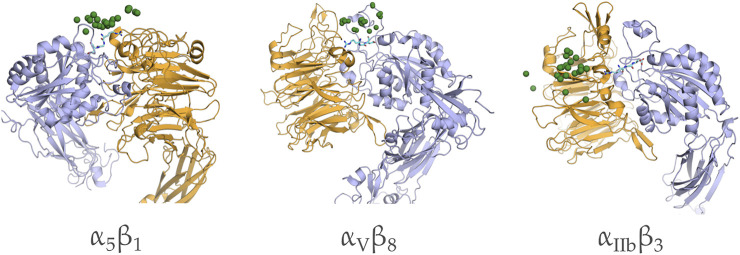
Distribution of the candidate complexes of RBD docked to *α*_5_ *β*_1_, *α*_IIb_ *β*_3_, and *α*_*V*_ *β*_8_ integrins. The positions of G405 of the RBD motif are shown in green spheres and the native bound configuration of RBD from the crystal structure is shown in cyan sticks.

We used 22 structures of the highly populated cluster centers obtained from the molecular dynamics simulation to conduct a protein-protein docking. The analysis was conducted by restraining the sampling space to include the RGD motif of RBD and the native binding site on *α*_5_ *β*_1_, *α*_IIb_ *β*_3_, and *α*_*V*_ *β*_8_ integrins (Figure 5). These integrins have been chosen mainly for their high-quality crystal structures in a bound state with an RGD motif. Of note, the homology relationship with RGD-binding integrins expressed in airway epithelial cells; namely *α*_*V*_ *β*_5_, *α*_*V*_ *β*_6_, and *α*_*V*_ *β*_8_, is confirmed, implying a conserved 3D fold. Moreover, *α*_IIb_ *β*_3_ was included to assess the putative binding of SARS-CoV-2 to platelets as suggested by previous studies (Koupenova and Freedman, 2020; Zaid et al., 2020; Zhang et al., 2020). Our results show that RBD has not been able to interact favorably with any of the studied integrins. Indeed, RGD motif was not capable of reaching its native binding site in any given structural state.

## 4 Discussion

The optimal interaction of the RGD motif with integrin involves the establishment of a minimal set of contacts with the MIDAS interaction site and the nearby amino acid residues. Experimental structures of RGD in the bound form with integrins show that the motif is laid extensively, crossing the interface cleft between the alpha and beta integrin subunits. The carboxylic and guanidine groups of RGD act as electrostatic clamps with the MIDAS site and the acidic residues of the alpha subunit respectively. However, when we superposed the RGD motif from the RBD domain of SARS-CoV-2 with its corresponding sequence on the cilengitide molecule co-crystallized with the integrin (data not shown), we found that severe clashes persist in this mode of interaction. Following this observation, we hypothesized that the RBD must undergo structural adaptation to allow for the favorable interaction with integrins.

The RMSIP distribution demonstrated that the conformational space sampled from the analysis of all the experimental structures are relatively homogeneous, given the observed low variance in the data. Therefore, it is expected that the normal mode properties are linked directly to the conformational behaviour of the RBD. Both normal mode analysis and molecular dynamics simulation are supportive of the relative rigidity of the RGD motif, compared to the RBM amino acids. Therefore, the motif is highly unlikely to undergo a significant structural rearrangement to increase its exposure to the solvent and allow the interaction with integrins. The RGD motif in the structure of different disintegrins, like triflavin, schistatin, echistatin, decorsin and salmosin is located at the tip of a hairpin-like structure that allows an easy fitting with the integration head cleft without steric hindrance (Matsui et al., 2010). The same type of structure was observed in *α*_*V*_ *β*_6_ integrin interacting with the capsid protein VP1 of the foot-and-mouth disease virus (Kotecha et al., 2017). In the case of SARS-CoV-2 RBD, the RGD motif did not show any structural similarities with disintegrins, and the steric hindrance imposed by the segments close to the motif, seems to be maintained in all the functionally relevant conformational states.

Microscale aMD allowed for an extensive sampling of the conformational phase of RBD where we have detected three highly populated states that could correspond to the bound, up and down configurations of the domain. However, potential integrin-binding conformations were not detected. The free energy landscape also confirmed that the geometrical features of the RGD binding to integrins are unfavorable. Moreover, protein-protein docking showed the inability of all the highly populated conformations to reach the depth of the interaction site of integrins where the electrostatic clamping and the interaction with MIDAS must happen to maintain a stable association.

Most of the former works have relied on sequence conservation and motif detection analysis to conclude on the implication of RGD motif in SARS-CoV-2 RBD in the interaction with integrins (Luan et al., 2020; Sigrist et al., 2020; Carvacho and Piesche, 2021; Dakal, 2021). However, few of them have considered the structural features to reinforce or confirm the hypothesis with details, as presented in this study. Indeed, Sigrist et al. (2020) and Luan et al. (2020), stated the solvent exposure of RGD as the single argument supporting its involvement in integrin binding, but they did not consider the geometrical features of the motif that must be fulfilled nor the steric hindrance that can be imposed by the surrounding segments. Mészáros et al. (2021) and Makowski et al. (2021) proposed that the surrounding residues of RGD are flexible and, therefore, allow the interaction with integrins. Nevertheless, our results from molecular dynamics simulation and normal mode analysis are congruent in showing that the level of plasticity of these segments is not sufficient to eliminate sterical hindrance that prevents the association with integrins. Moreover, we were not able to detect any hairpin-like structure as observed in disintegrins and VP1 protein of the foot-and-mouth disease virus, despite the extensive sampling of the conformational space. Computational analysis by Dakal (2021) concluded that the RGD could bind favorably to *α*_5_ *β*_1_ and *α*_5_ *β*_6_ integrins. However, in his study the author used only the *β*-propeller head of the alpha subunit for the protein-protein docking, which is not adequate to infer physiological binding properties. On the other hand, Beddingfield et al. (2021) showed that the protein-protein complex between integrins and S protein, obtained from docking, does not show a favorable fitting in the RGD binding site, which is in agreement with what we have observed from the constrained protein-protein docking analysis.

Among integrins expressed in airway epithelial cells, and that could be potential SARS-Cov-2 recepteor, *α*_2_ *β*_1_, a collagen and laminin receptor, plays a critical role in cell infection by echovirus (Eisner, 1992). The *α*_2_ *β*_1_ integrin is known to be a non-RGD binding receptor, and therefore, it is unlikely that it binds to the 403–405 segment of RBD. The second receptor *α*_*V*_ *β*_5_ is well known to be an adenovirus receptor (Wickham et al., 1994), but is not expressed on the luminal surface (Grubb et al., 1994) which makes it difficult to be involved in the infection by coronavirus. *α*_*V*_ *β*_6_, an RGD receptor, was described to be implicated in infection by foot and mouth disease virus (Jackson et al., 2000). *α*_*V*_ *β*_6_ is the only one known to be expressed on the mucosal epithelial cells that are the primary site of infection by respiratory viruses (Sheppard, 2003). However, studies using developed antibodies show that *α*_*V*_ *β*_6_ is poorly expressed in lung epithelium cells and is constitutively expressed at low levels in uninjured epithelia (Breuss et al., 1995; Weinacker et al., 1995). Furthermore, the expression pattern of RGD-binding integrins is very differentiated between healthy and unhealthy pulmonary cells. Indeed, many integrins are not seen on healthy adult airway epithelium cells especially *α*_5_ *β*_1_ and *α*_9_ *β*_1_ (Pilewski et al., 1997; Sheppard, 2003). On the other hand, the other expressed RGD dependent integrins have a distinct functional, spatial and chronological expression (Pilewski et al., 1997). *α*_*V*_ *β*_5_, *α*_*V*_ *β*_6_ and *α*_*V*_ *β*_8_ are constitutively expressed at low levels on healthy lung cells (Breuss et al., 1995; Sheppard, 2003), recognize many ligands that are not expressed on healthy epithelial basement membranes, and are only involved in cases of lung inflammation and injury (Nishimura et al., 1994; Nishimura et al., 1994; Mu et al., 2002). Nader et al. (2021) have conducted experiments to assess the binding of SARS-CoV-2 spike protein to integrin *α*_*V*_ *β*_3_, to Human Aortic Endothelial Cell or to Caco-2 endothelial cells. Their result shows indeed a direct interaction with the integrin. However, a competition assay with Cilengitide, an RGD binding peptide, was only conducted for the cell binding assay. It is therefore difficult to assert whether the observed effect in their work is the result of the direct interaction of the RBD-RGD motif with avb3 or if it is the outcome of a modulation effect. It is worthy to note, that the Cilengitide can induce the downregulation of the *ITGAV* gene which encodes the *α*_*V*_ subunit (Wang et al., 2014). On another note, Schimmel *et al.* demonstrated that primary endothelial cells can not be infected with SARS-CoV 2 *in vivo* nor *in vitro* (Schimmel et al., 2021). Moreover, another study has concluded that the incubation with the integrin inhibitor ATN-161, had no effect on the infection capacity of SARS-CoV-2 with Caco-2 endothelial cells (Zech et al., 2021). All these studies are not contradicting our results. In fact, we are not excluding integrins as putative receptors for SARS-CoV-2. We, however, postulate that RGD from RBD is unlikely to be the interacting motif with integrin. This implies that other motifs could be involved in such interaction. Our claim, is also sustained with the recent study by Beaudoin et al. (2021). All this information, consolidated by our above-cited results, emphasize the need for more evidence to confirm the role of integrins in the physiopathology of SARS-CoV-2.

## 5 Conclusion

Based on the evidence provided in this paper, we suggest that the RGD motif from the RBD of SARS-CoV-2 is unlikely to interact with integrins. That, however, does not imply that integrins are not host receptors for the virus. Thus, in light of our results, as well as previous works, the potential interaction of the RGD motif from the RBD of SARS-CoV-2 with integrins should be revised extensively. Consequently, potential involvement of other segments belonging to the spike protein, is more likely to take place if integrins are confirmed to be host receptors for SARS-CoV-2.

## Data Availability

Molecular dynamics trajectories, raw data and the code used to make the analysis and figures of this paper are available online from the Zenodo repository “*Dry trajectories of SARS-CoV-2 RBD from accelerated molecular dynamics simulation*” https://doi.org/10.5281/zenodo.5775514.
